# Vaccination with *Salmonella* Typhi recombinant outer membrane protein 28 induces humoral but non-protective immune response in rabbit

**DOI:** 10.14202/vetworld.2017.946-949

**Published:** 2017-08-19

**Authors:** Anjani Saxena, Rajesh Kumar, Mumtesh Kumar Saxena

**Affiliations:** 1 Department of Veterinary Biochemistry and Physiology, College of Veterinary and Animal Sciences, G.B. Pant University of Agriculture and Technology, Pantnagar, Uttarakhand, India; 2 Department of Veterinary Microbiology, College of Veterinary and Animal Sciences, G.B. Pant University of Agriculture and Technology, Pantnagar, Uttarakhand, India

**Keywords:** adjuvant, outer-membrane proteins, recombinant protein, *Salmonella*, vaccine

## Abstract

**Aim:**

Typhoid is one of the most important food and water borne disease causing millions of deaths over the world. Presently, there is no cost effective vaccine available in India. The outer-membrane proteins (Omps) of *Salmonella* have been exhibited as a potential candidate for development of subunit vaccine against typhoid. The objective of the present study was to evaluate the use of recombinant Omp 28 protein for immunization of rabbit to elucidate its protection against virulent *Salmonella* Typhi.

**Materials and Methods:**

Immune potential of recombinant Omp28 was tested in New Zealand Rabbits. Rabbits were divided into two groups, i.e., control and test group. Control group was injected with phosphate buffer saline with adjuvant while test group were injected with recombinant Omp28 along with adjuvant. Rabbits were bleed and serum was collected from each rabbit. Serum was tested by Enzyme-linked immunosorbent assay (ELISA) for humoral response. Rabbits were challenged with virulent culture to test the protective immunity.

**Results:**

Humoral response was provoked at 15^th^ day and maintained till 30^th^ day. The mean ELISA titer at 15^th^ day was 1 : 28000 (mean titer log 10 : 4.4472) and on the 30^th^ day was 1 : 25866 (mean titer log 10 : 4.4127). Protective immune potential of Omp 28 was assessed by challenge studies in rabbits for which vaccinated and control rabbits were challenged with 10^9^ cells of virulent culture of *S*. Typhi. In control group, out of six, no rabbit could survive after 48 days while in vaccinated group, three out of six rabbit were survived.

**Conclusion:**

Immunization of rabbit with recombinant Omp 28 induced a strong humoral response which was exhibited by high antibody titer in ELISA. Subsequently, intraperitoneal homologous challenge of the immunized New Zealand rabbit resulted in lack of significant protection. These findings indicate that Omp 28 though provoked the humoral immunity but could not provide the protective immunity in rabbit model.

## Introduction

Typhoid fever is a public health problem with an estimated 22 million cases and 220,000 related deaths occurring worldwide each year [1]. It is a life-threatening illness caused by *Salmonella* enterica serovar Typhi. With the advancement of molecular biology polymerase chain reaction (PCR)-based techniques have been extensively used for detection and differentiation of pathogens [2-6] which had made possible to detect the disease in early stages but the development of resistance to antimicrobial agents and the possible reversal of the resistance in *S*. Typhi has become a significant issue leading to difficulties in the management of disease [7,8]. Vaccination against *S*. Typhi is an essential tool for the effective management of typhoid fever. Presently available typhoid vaccines have several limitations such as short term immunity, and they are not cost effective [9]. Therefore, there is a need for better and improved new generation vaccine against *S*. Typhi.

The outer-membrane proteins (Omps) are conserved protein of Gram-negative bacteria and have immune reactivity. Several Omps had been isolated and targeted as an antigen and few of them had exhibited their immune potential [10,11]. However, it is very suggestive to purify a single Omp for commercial vaccine production as it is not cost effective. To overcome from this problem recombinant DNA (rDNA) technology had been used for bulk production of desired protein. Several Omps had been target as a candidate for r-DNA vaccine against typhoid [11,12]. Omp 28 is which had been purified from *Salmonella* Typhi. It was further sequenced, and few tests had been conducted to assess its immune potential in which it had exhibited promising potential [13,14]. Therefore, in our earlier study, we targeted Omp 28 as rDNA vaccine against *S*. Typhi.

We had cloned, sequenced and mapped major epitopes (B and T epitopes) of Omp 28. Our preliminary study on the basis of bioinformatic analysis indicated that Omp 28 can provoke humoral as well as cell mediated immunity [15]. Similar type of findings was reported in the case of *S*. Typhimurium [16]. Therefore, we expressed Omp 28 and in the present study tested the immune potential of Omp 28 in rabbit model to explore the possibility of development of Omp 28 based subunit vaccine against *S*. Typhi.

## Materials and Methods

### Ethical approval

The ethical approval for this work was accorded by Institutional Animal Ethics Committee of G.B.P.U A&T Pantnagar.

### Bacterial strains and vectors

The culture of *Salmonella* Enterica subsp. enterica serovar Typhi MTCC 733 was procured from Institute of Microbial Technology (Chandigarh, India) and grown on LB agar. *Escherichia coli* strain DH5α used in the cloning experiments was purchased from Bangalore Genei (India) and grown in Luria broth. The *E. coli* M_15_ cells were used for the expression of recombinant protein, and pQE 30 expression vector was procured from Qiagen (USA).

### Animals

New Zealand rabbit of 6 weeks was procured from Central Drug Research Institute (CDRI), Lucknow, Uttar Pradesh, India. Animals were certified as disease and pathogen free by CDRI, Lucknow, Uttar Pradesh, India. 12 rabbits were used in vaccine trial. Animals were reared as per the ethical guidelines approved by Institutional Animal Ethics Committee of the university. Animals were housed in small animal house of the department and provided the recommended feed and water. Before vaccination, all the rabbits were bleed by ear veins under aseptic conditions. Serum was collected and tested for pre-existing antibodies against *Salmonella* by double immune diffusion test with *Salmonella* Omp antigen. Animals were also tested by cloacal swab culture for the presence of *Salmonella*.

### Vaccination and trial studies in rabbit

The rabbits were divided into two equal groups. The first group of six rabbits was taken as control and injected with adjuvant in phosphate buffer saline (PBS). The other group, test group of six rabbits was used for vaccination with r-Omp 28 antigen. Test group was injected subcutaneously with 100µg purified recombinant protein with equal amount of adjuvant, followed by two boosters at 21^st^ and 42^nd^ day post vaccination. Rabbits were bleed 15^th^ day after last booster. But no significant titer was observed. Therefore, two more boosters of 500 µg with equal amount of adjuvant were injected at 15 day intervals. Controls were also injected with same volume of PBS with adjuvant with same schedule.

### Collection of sera

Rabbits (control and vaccinated) were bled through ear veins on the 15^th^ and 30^th^ day after last booster and blood were collected in sterile microfuge tubes. The collected blood samples were kept at room temperature for an hour and further kept at 4°C for retraction. Finally, the serum was separated and kept at −20°C till further use.

### Humoral immune response

Sera collected from rabbits at 15^th^ and 30^th^ day were used for assessment of humoral immune response by indirect enzyme-linked immunosorbent assay (ELISA) [17]. 96 well ELISA plates were charged with 100 ng/well protein and incubated overnight at 4°C. Plates were washed with Phosphate buffer saline with tween (PBST) 0.5% twice to remove unbound antigen and blocked with 2% bovine serum albumin for 1 h. Serum from each rabbit was diluted and used in duplicate. Plates were incubated at 37°C for 2 h followed by three washing with PBST to remove unbound primary antibodies. After three washings 100 µl secondary antibodies (anti rabbit HRP conjugate) in dilution of 1:3000 were poured in each well. Plate was incubated at 37°C for 2 h, followed by three washing with PBST. Substrate ortho phenyl diamine was added, and after the development of color OD was taken at 450 nm (substrate buffer: 0.1 M citric acid solution, 0.2 M dibasic sodium phosphate solution, and ortho phenyl diamine 40 mg, H_2_O_2_ 40 µl). Dilution at which OD of vaccinated group was more than twice of control group was considered the titer of ELISA. Mean optical density at the dilution of 1:25600 of test and control group was compared to determine the efficacy of humoral response. To assess the protective immune response of vaccine 4 weeks after the last booster dose rabbits were challenged with 10^9^ CFU (I/P) of virulent strain of *S*. Typhi [18]. Mortality as observed till 48 h of challenge.

### Statistical Analysis

Humoral response in vaccinated and control group at different time interval was analyzed using unpaired t-test, and the mean titer was calculated by deriving the mean of logarithmic value of reciprocal of titer with standard error. The results of challenge studies were analyzed using Chi-square test with Yate’s correction.

## Results and Discussion

### Vaccination trial and humoral response to vaccine

**Figure-1:**
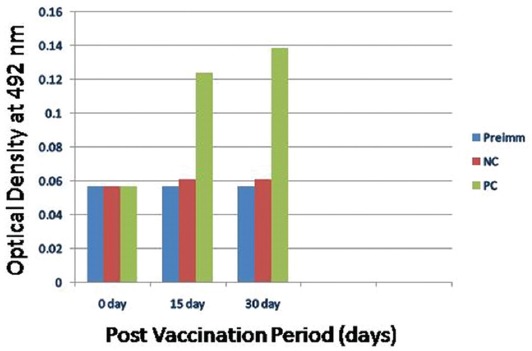
Mean absorbance values of the humoral immune response in rabbits evaluated at different time interval at dilution 1:25600. Preimm: Pre immunized serum, NC: Negative control, PC: Positive control.

The humoral response in mice was analyzed by indirect ELISA test. Humoral response was provoked at 15^th^ day and maintained till 30^th^ day. The mean ELISA titer at 15^th^ day was 1 :28000 (mean titer log10 : 4.4472) and on the 30^th^ day was 1 : 25866 (mean titer log 10 : 4.4127). The mean absorbance value of control and vaccinated group was compared at the dilution of 1:25600 (Figure-1) it was found to be 0.061 and 0.1241 on 15^th^ day and 0.061 and 0.1391 on 30^th^ day respectively. Humoral response in vaccinated and control group at different time interval was analyzed using unpaired t-test which was maintained till 30^th^ day.

Protective immune potential of Omp 28 was assessed by challenge studies in rabbits for which vaccinated and control rabbits were challenged with 10^9^ cells of virulent culture of *S*. Typhi [18]. In control group, out of six, no rabbit could survive after 48 days while in vaccinated group, three out of six rabbit were survived. The findings indicated that though the mortality was reduced after the vaccination with Omp 28 but Omp 28 could not provide complete protective immunity. First report on Omp 28 indicated that the level of antibodies against Omp 28 was very high in serum of collected from patients of typhoid [13]. Further, mice antibody raised against Omp 28 in mice showed anti-salmonella activity [14] *in vitro*. These results indicated the immunogenic importance of Omp 28 isolated from *Salmonella*. Therefore, Omp 28 was selected for the study. Many other workers had also tested recombinant Omps such as Omp A, Omp C, Omp F, Omp S_1_, and Omp S_2_ for their immunogenic potential. Among these proteins, Omp A, Omp C, and Omp F had exhibited immunogenicity [19] but could not achieve complete protection.

Two porins Omp S_1_ and Omp S_2_ from *S*. Typhi could produce of long term antibody titer and complete protection against *S*. Typhi challenge [20]. Porins Omp S_1_ and S_2_ induced tumor necrosis factor interleukin (IL)-6 and IL-10 production. Omp S_1_ and Omp S_2_ despite being expressed at low levels under *in vitro* culture conditions proved as potent protective immunogen with intrinsic adjuvant properties. Similarly, Omp L had also been tested in mice model and had shown a promising protective immunity [21]. It was highly immunogenic and provoked humoral and cell mediated immune response. It conferred 100% protection to immunized mice against *S*. Typhi challenge studies. PagN an Omp of *S*. Typhimurium as a potential vaccine candidate for salmonellosis. PagN has also been cloned, expressed and tested for its immune potential in mice and considered as a target molecule for the development of rDNA vaccine against *S*. Typhi [11].

An adhesion protein of *S*. Typhi named as T 2544 as potential targeted for vaccine development [22]. It had produced a high titer of IgG and IgA and strongly immunogenic and proved as potential vaccine candidate. Similarly, recombinant Omp C protein of *Salmonella* has been tested in birds for immunization of birds to elucidate its protection against virulent *S*. Typhimurium [23]. They reported r-Omp C induced a significantly high humoral immune response with a stable cell mediated immune response. Besides immune response a protective index ranged between 50% and 75% has also been observed for 3 weeks after challenge. The findings of earlier workers and our study indicate that several Omps have been targeted for development of rDNA vaccine against *Salmonella*. Some of them such as Omp S1, Omp S_2_, Omp L, and PagN have exhibited the promising potential, but some other proteins such as Omp A, Omp F, and Omp 28 could produce only partial protection. Therefore, there is a need to evaluate the effect of combination of two or more Omps along with advanced adjuvant system [24] to develop a safe and effective vaccine against *S*. Typhi.

## Conclusion

Immunization of rabbit with recombinant Omp 28 induced a strong humoral response which was exhibited by high antibody titer in ELISA. Subsequently, intraperitoneal homologous challenge of the immunized New Zealand rabbit resulted in lack of significant protection. These findings indicate that Omp 28 though provoked the humoral immunity but could not provide the protective immunity in rabbit model. Therefore, a combination of more than one Omp may be targeted to achieve complete protective immunity.

## Authors’ Contributions

AS and RK had performed the whole experiment while MKS being the mentor of the project designed the experiment. All authors read and approved the final manuscript.
